# Integration of ultrasonography training into undergraduate medical education: catch up with professional needs

**DOI:** 10.1186/s13244-022-01296-3

**Published:** 2022-09-24

**Authors:** Ulf Teichgräber, Maja Ingwersen, Claudia Ehlers, Hans-Joachim Mentzel, Christoph Redies, Andreas Stallmach, Wilhelm Behringer, Orlando Guntinas-Lichius

**Affiliations:** 1 Faculty of Medical Education, Friedrich Schiller University, Jena University Hospital, Am Klinikum 1, 07747 Jena, Germany; 2 Department of Radiology, Department of Diagnostic and Interventional Radiology, Friedrich-Schiller-University, Jena University Hospital, Am Klinikum 1, 07747 Jena, Germany; 3 Institute for Anatomy, Friedrich Schiller University, Jena University Hospital, Teichgraben 7, 07743 Jena, Germany; 4 Department of Internal Medicine IV, Friedrich Schiller University, Jena University Hospital, Am Klinikum 1, 07747 Jena, Germany; 5 Department of Emergency Medicine, Friedrich Schiller University, Jena University Hospital, Am Klinikum 1, 07747 Jena, Germany; 6 Department of Ear, Nose and Throat Medicine, Jena University Hospital, Am Klinikum 1, 07747 Jena, Germany

**Keywords:** Curriculum, Professional competence, Ultrasonography, Undergraduate medical education

## Abstract

**Objective:**

Ultrasonography (US) has become the first-line imaging modality even for physicians who are not imaging specialists. The progress has not yet been sufficiently considered in medical education. The aim was to develop a curriculum that integrates US as a compulsory part into medical education directly from the start, to build up professional competencies toward residency.

**Methods:**

Development was based on Kern’s six-step approach to identify problems, specify needs, define goals, outline strategies, and propose methods.

**Results:**

The proposed curriculum follows a spiral course within which students should pass through four levels of training with increasing complexity. Students will be asked to independently prepare for courses by using learning videos. On the first training level, US should be closely linked to anatomy and physiology courses. Competency-centered courses should be held in small groups. On the second level, in the third year of education, students will apply point-of-care ultrasonography concerning multiple medical disciplines. On the third level, they will select a compulsory course in a specialty of their choice, held at five consecutive dates. From then on, US will be conducted in patients. Finally, during the final year, students are expected to use US under pro-active supervision with a large degree of independence and confidence. Throughout the curriculum, the discipline of radiology combines vertically with foundational sciences and horizontally with other medical specialties.

**Conclusion:**

The conceptual proposal for a longitudinal US curriculum presented here has been developed by radiologists to equip students with competencies needed for contemporary patient care.

**Supplementary Information:**

The online version contains supplementary material available at 10.1186/s13244-022-01296-3.

## Key Points

- The proposed curriculum includes US training in four levels of difficulty.
- Early and continuous “probe-in-the-hand” US training in small groups covers multiple disciplines.
- Graduates will use ultrasonography with a large degree of independence and confidence.

## Introduction

In the last decades, diagnostic ultrasonography has emerged as primary diagnostic approach across most medical specialties. At the same time, equipment improved and nevertheless remains affordable. Ultrasonography is noninvasive and thus can be done immediately and repeatedly without exposing patients to radiation [1]. No transport to computed tomography or magnetic resonance imaging suites is required [2]. Therefore, point-of-care ultrasonography (POCUS) is used beyond the circle of radiologists in various clinical settings. Medical education should be adapted to these changing opportunities and requirements.

Due to complexity and multi-disciplinarity, ultrasonography (US) cannot be trained in a short course, and competency cannot be measured at one point. Considering the World Federation of Ultrasound in Medicine and Biology (WFUMB) perspective [3], we therefore propose to introduce US training early and to provide a longitudinal curriculum with major contribution of the discipline of radiology that follows a spiral path throughout the entire period of medical education. Requirements to be placed on the students and complexity of repetitive topics should increase over time, starting with handling of the ultrasonography probe, over imaging anatomy and recognition of appropriate indications, until recognition of pathologies. According to the recommendation of the European Federation of Societies for Ultrasound in Medicine and Biology (EFSUMB), US training should be vertically integrated into preclinical and clinical specialties [4]. Self-determined learning [5, 6] and “probe-in-the hand” training in small groups should be basis elements. Selected entrustable professional activities (EPAs), to which learners should be enabled at supervision levels that correspond to the respective stages of training, are defined as intended learning outcomes (ILOs). During the curriculum, for entry into residency, students should acquire professional competence to conduct ultrasonography as a “whole” in general medical and emergency clinical indications.

**Fig. 1:**
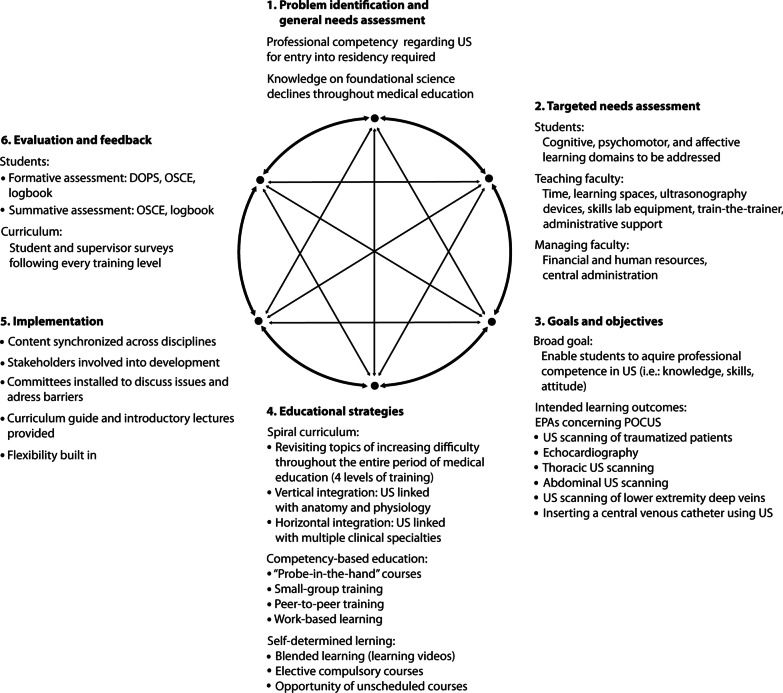
Six step approach of curriculum development according to Kern [7] adapted for ultrasonography in medical education. EPA = Entrustable professional activity, DOPS = direct observation of procedural skills, OSCE = objective structured clinical examination, POCUS = point-of-care ultrasonography, US = ultrasonography

Our purpose was to develop a longitudinal ultrasonography curriculum according to the model of Kern [7] considering the aspects of problem identification, targeted needs, goals and objectives, educational strategies, implementation, and evaluation (Fig. 1). We provide a conceptual proposal.

### Problem identification and general needs

Although ultrasonography is a user-dependent technology [1] and both technique and interpretation require some practice, demand for training is not yet reflected in medical education. To date, in most medical schools, students experience their first encounter with ultrasonography only in their third year of medical education [8]. In the USA, 62% of medical schools integrate ultrasonography training into undergraduate medical education but only 19% give high priority to it [8]. In Europe, only 17% of universities integrate ultrasonography into anatomy courses. (A summary overview of established curricula is shown in Additional file 1: Table S1.) Average number of ultrasound lessons is reported with seven. Main reasons for not having ultrasonography integrated into medical education are lack of time as well as poor faculty and equipment funding [3, 8, 9]. High expense for most parts can be explained by requirement of small-group training. Nonetheless, time is running out for students to sufficiently practice skills required from general practitioners.

In parallel, from students’ point of view, relevance of foundational sciences, such as anatomy and physiology, remains vague and acquired knowledge declines throughout advanced clinical education. As a result, basic knowledge is not fully available where it is needed, namely in medical imaging. We assume that the earlier students start performing ultrasonography with “probe-in-the-hand” training, and the more consistent they grow into more complex clinical ultrasonography practice, the easier they will handle it as an integral part of patient care. Therefore, we developed a spiraling integrated curriculum which may serve as a pilot program for other clinical methods that require training of complex professional tasks.

### Targeted needs

To improve competency, change is needed in both content and strategy of ultrasonography education. ILOs have to be defined and constructively aligned to learning activities and assessment [10]. In this respect, the program leader (preferably the department of radiology) should consider key issues of stakeholders including students, teaching staff, and faculty management during development and maintenance of the curriculum.

Key issue of students is that all three domains of learning, i.e., cognitive, psychomotor, and affective, are to be addressed at all levels of medical education to promote increase and preservation of knowledge as well as development of skills and attitude. The WFUMB recommends at least 40 h of practical ultrasonography training during early years of medical education [3]. Key issue of teaching staff and faculty management is to coordinate and synchronize basic and clinical science lectures and courses regarding content and timing with affiliated ultrasonography courses. Senior faculty members of all participating departments can be involved in selection, content validation, and assessment of ILOs. Teaching staff should be provided with resources required including time, learning spaces, ultrasonography devices, skills laboratory simulators, and administrative support. Schedule conflicts of practicing clinicians should be anticipated as far as ahead as possible. To reduce personnel time and costs, senior students can be involved in tutoring [3, 11]. In addition, advice from senior students, the so-called near-peer teaching improves learning in both senior and junior students. Cognitive and social congruence of peers might foster learning [12]. However, student tutors will need extended education including didactical content [11] provided by the department of radiology. Faculty management should agree to ILOs, use of financial and human resources, and responsibilities regarding student assessment and apportionment between central and departmental administration.

### Goals and objectives

The ultrasound curriculum will accompany learners throughout their entire medical studies. Broad goal is to achieve continuously growing competence, i.e., knowledge, skills, and attitude regarding use and limitations of the complex professional task of ultrasonography.

The goal shall be reached through learning activities addressing revisiting topics of increasing complexity each relating to preceding learning steps [13]. During preclinical education, measurable objectives of course attendance are image acquisition and identification of specified anatomical structures and physiological processes using ultrasonography. Building on this, 3rd-year learners will participate in POCUS courses, which reflect selected relevant professional requirements. At that time, ILOs are specified as competency frameworks in a set of six entrustable professional activities (EPAs) corresponding to the respective POCUS tasks. EPA development process is presented below. Core ultrasonography EPAs of the curriculum are as follows (detailed description of EPAs is provided in Additional file 1: Tables S2–S7):

1. Ultrasonography scanning of traumatized patients
2. Echocardiography
3. Thoracic ultrasonography scanning
4. Abdominal ultrasonography scanning
5. Ultrasonography scanning of lower extremity deep veins
6. Inserting a central venous catheter using ultrasonography guidance

EPAs specify required knowledge, skills, attitudes, and experiences not only to allow summative entrustment but also to guide learners toward progressively decreasing supervision and to prepare assessors [14]. Knowledge and skills are EPA specific, and requirements increase with number and complexity of experiences. Thus, expectations placed on 3^rd^-year students who only had the opportunity to perform ultrasonography on peers or simulators are lower compared to expectations on advanced students particularly in the final year. EPAs should be trustworthily conducted under direct supervision for entering the final year and under indirect supervision for entering residency.

In contrast to knowledge and skills, attitudes are generally applicable. Overall, Kennedy et al. [15] and ten Cate et al. [14] proposed five types of general features that are critical to establish trustworthiness in learners: agency, reliability, integrity, capability, and humility. With respect to attitudes required for EPAs, patient’s experience should be considered most relevant [14].

### Educational strategies

Overall, the proposed ultrasound curriculum is structured as spiral curriculum consisting of four levels of training with the characteristic feature of revisited topics of increasing difficulty, each related to the preceding level [13]. Vertical integration provides a connection of foundational with applied sciences—in our case of anatomy and physiology with radiology. Thereby, basic science is brought into context and vice versa, clinical aspects are introduced into medical training [16, 17]. Utility of basic science will become obvious to students—an important issue to motivate adult learners. In addition, horizontal integration combines different medical specialties [18]—in our case different clinical sciences with radiology. Simultaneously, the curriculum intends to permanently encourage the three domains of cognitive, psychomotor, and affective learning using the concepts of blended and competency-based learning.

Blended learning [5, 6] shall be implemented by providing students with maximum 10 min. long video lectures so that they can prepare themselves for ultrasonography courses. Beforehand, faculty should prepare appropriate videos to demonstrate learning activities that correspond to the actual level of training. Videos will be kept permanently available online for the students via university content management system. Previously, video lectures had been shown to be equally effective as conventional lectures in preparation of clinical examinations. A side benefit of blended learning is to reduce the need of teachers for lectures and thus decrease financial burden. Additionally, learning material to prepare, reflect, and deepen knowledge is, for example, available on the EFSUMB e-learning platform [4]. All in all, we rely on students to prepare themselves for sonography courses in a self-determined manner. Finally, self-determined learning might prevent overloading of students with excessive information.

Hands-on training is based on competency-based medical education [19]. Students will start to practice ultrasonography by scanning each other, attend training in skills laboratories, and, finally, deepen experience by scanning patients in clinical settings. Thereby, learning activities will gradually be placed into clinical context (Table 1).

**Table 1:** Proposed compulsory longitudinal ultrasonography curriculum

	Educational strategies	Objectives	Assessment
Entire study period	• Spiral curriculum: revisiting topics of increasing difficulty	• Knowledge	Summative and formative
	• Content synchronized across disciplines	• Skills	
	• Flexibility built-in	• Attitude	
Preclinical (US training level 1)	US courses in conjunction with anatomy dissection and physiology courses (2 teaching units* per topic)	Topics:	DOPS (1 per topic)
		• Shoulder girdle, rotator cuff	
	• Students prepare themselves with learning videos	• Thoracic image acquisition	
	• “Probe-in-the-hand”	• Abdominal image acquisition	
	• Small groups (students examine each other)	• Carotid artery, jugular vein, thyroid gland, neck muscles	
	• Opportunity of unscheduled courses	• Doppler sonography: echocardiography, carotid artery	
Clinical, third year (US training level 2)	POCUS, small groups (1 teaching unit per EPA)	EPAs:	DOPS (1 per EPA)
	Skills laboratory, simulation (1/2 teaching unit* per EPA, peer-to-peer)	• Sonography for trauma	OSCE
	• Students prepare themselves with learning videos	• Echocardiography	
	• “Probe-in-the-hand”	• Thoracic ultrasonography	
	• Small groups (students examine each other/simulation)	• Abdominal ultrasonography	
	• Opportunity of unscheduled courses	• Lower extremity deep veins	
		• US-guided central venous access	
Clinical, advanced (US training level 3)	Elective compulsory course (10 teaching units* distributed to 5 dates)	Options (one of the following):	DOPS (at least 2 per EPA)
	• Students prepare themselves with learning videos	Radiology, gastroenterology, cardiology, general surgery, orthopedics, gynecology, pediatrics, urology, otolaryngology	
	• Small groups (US scanning on patients)		
Sub-internship, final year (US training level 4)	Work-based learning (supervised “on-field” experience)	Broad range of clinical settings (in- and outpatient care)	DOPS
			Logbook

^*^A teaching unit equals 45 min. DOPS = direct observation of procedural skills, EPA = entrustable professional activity, OSCE = objective structured clinical examination, POCUS = point-of-care ultrasound, US = ultrasound

The 1st level of curricular ultrasonography training is to be passed through the preclinical stage of medical education. Established anatomy dissection courses on four specified topics will each be linked to one and a half hour “probe-in-the-hand” ultrasonography courses. In addition, students should complete a physiology hands-on course on Doppler ultrasonography covering echocardiography and imaging of the carotid artery. Responsible faculty will coordinate courses in both substance and timing. Students should conduct ultrasonography examination on each other in small groups with a maximum of 6 learners under supervision of the tutor. The multidisciplinary team of supervisors will consist of anatomists, physiologists, radiologists, practicing clinicians, and student tutors. Vertical linkage of anatomy and ultrasonography is supposed to support training of spatial and figurative imagination needed for cross-sectional imaging techniques and thus, improve psychomotor skills. However, synchronous presentation of content and material will pose logistical challenges to faculty.

On the 2nd level of training, in the third year of students’ medical education, POCUS should be practiced in five different disciplines. POCUS builds upon students’ earlier preclinical experiences with ultrasonography and consists of one or more focused examinations to diagnose or rule out specific conditions in real-time or to provide procedural guidance. An earlier study reported that diagnostic accuracy of cardiovascular POCUS conducted by students was superior to physical examination alone by cardiologists [20]. The proposed curriculum will include POCUS in the fields of emergency medicine, cardiology, pulmonary medicine, gastroenterology, and angiology, as well as POCUS to guide insertion of a central venous catheter. ILO of every POCUS is specified as corresponding EPA as elaborated before (Additional file 1: Tables S2–S7). Every POCUS course will last 45 min and will be supervised in small groups of maximum 6 students. Again, students should conduct ultrasonography in peers. Image acquisition and identification of organ structures are paramount. Anatomists and physiologists will still accompany learners as supervisors to facilitate understanding of pathology and pathophysiology and to provide a persistent but decreasing revisit of basic science. In parallel, corresponding to every POCUS course, a 30-min ultrasonography skills laboratory training is provided in which students should run through simulations of pathological findings and conduct an ultrasonography-guided central venous cannulation on a training phantom. Skills laboratory activities will solely be supervised by student tutors. Skill laboratories will be available during semester breaks to provide the opportunity to practice outside scheduled courses [21]. The POCUS approach requires coordination of times across departments. However, despite integration of ultrasonography into multiple disciplines, certain boundaries between courses and departments might remain. Structured communication across supervisors from different departments might overcome compartmentalization concerning ultrasonography.

The 3rd level of the ultrasonography training shall be provided during advanced clinical education. Students will now participate in more comprehensive compulsory ultrasonography courses, however, “caught up” at their level of skills [3]. They will be asked to choose one out of a menu of nine course options (i.e., radiology, gastroenterology, cardiology, general surgery, orthopedics, gynecology, pediatrics, urology, and otolaryngology) to direct their own learning and to focus on their preferred specialty. Every course is made up of 5 dates at 90 min each. Again, courses are to be held in small groups. Under direct pro-active supervision, students should examine patients. Although large number of options attended by a few students can be expensive, individualization is one of the four principles of teaching (i.e., feedback, activity, individualization, relevance) and is supposed to increase motivation. Activity, another principle of teaching should be incorporated by involving students in the clinical setting of workplaces in their selected departments. As before, in addition to practicing clinicians, anatomists and physiologists will accompany students during their elective courses. In accordance with available studies, we expect this approach to enhance foundational knowledge rather than to decline as it used to be over the course of medical education.

On the 4th level of training, during the final year, learners will gain “on-field” experience through work-based learning [22]. They should be enabled to conduct ultrasonography for the most part independently. Learners are required to organize and recall information from previous levels and to successively adapt skills and knowledge to growing medical experience with actual patients. Logbooks including lists of skills and competencies to be demonstrated by students should serve to structure and standardize the training. Finally, availability of ultrasonography should not deter students from physical examination. Particularly, visual inspection and palpation are irreplaceable. Instead, findings should be correlated [23].

### Implementation

Integration of ultrasonography into preclinical and clinical sciences should comprise both logistical changes and content. Early experience in implementation of the US curriculum at our university hospital revealed initial resistance to organizational change from clinicians and administrative faculty. Therefore, our approach required numerous open discussions with the parties involved. Committed and supportive leadership and strong collaboration and communication behaviors were demanded. Only the students were enthusiastic from the start. It may be appropriate to start with limited number objectives and expand by and by. To accommodate the needs of the multidisciplinary faculty, the department of radiology provided 12 US units intended for the sole purpose of teaching. In addition, a central practice room and a skills laboratory were made available for all participating departments. Arguments to convince faculty of foundational sciences to join the US curriculum were the opportunity to network and exchange with clinical sciences, to share and thus, save resources, and to promote students to deepen and preserve their foundational knowledge. Faculty development for US education can be a welcome opportunity. In addition, US courses may replace lectures in part, when blended learning can be implemented. However, collaboration should be synchronized, and contents should be linked. Therefore, from the outset, participating departments need to be included in the development to address barriers such as resources and acceptance of the multidisciplinary. For this purpose, the program leader may set up two committees: first, the implementation committee consisting of the dean, the program leader, and a medical education research associate, and second, the content committee consisting of the program leader, attending physicians of participating departments, anatomists, and physiologists. Central administration should be encouraged to coordinate development, maintenance, evaluation, and revision of the curriculum. Overall, flexibility should build into the curriculum to manage changing needs.

EPA development process of the proposed curriculum included content validation through a modified two round online Delphi consensus procedure at our institution [24], preceded by a comprehensive literature research [2, 11, 14, 21, 25, 26]. The Delphi panel consisted of a team of 30 attending physicians of 12 medical specialties (i.e., anatomy, physiology; abdominal and general surgery, anesthesiology, otorhinolaryngology, emergency medicine, gynecology, internal medicine, orthopedics, radiology, and urology).

It is recommended to provide students and faculty with electronic and printed curriculum guides that include information on EPAs, curriculum timetable, learning opportunities and resources, support, and assessment. However, lessons learned from ongoing curricula suggest not to overwhelm learners with material [21]. Short introductory lectures into courses at the beginning of the curriculum and at the start of every level may be beneficial to communicate course format and ILOs in relation to the overall focus.

### Evaluation and feedback

ILOs are the six aforementioned EPAs with increasing entrustment level corresponding to the stage of training. Continuous assessment should be aligned to these objectives [27]. At all levels, students’ competencies will be assessed by direct observation of procedural skills (DOPS). DOPS serve as formative assessment. Supervisors are encouraged to provide direct, narrative feedback. Case-based discussions may complete assessment with DOPS. Successfully passed DOPS allow students to continue with their curriculum. In general, the focus on formative assessment supports the approach of competency-based and self-determined learning.

After completion of the 2nd level of ultrasonography training including POCUS and skills laboratory courses, students must pass the regular objective structured clinical examination (OSCE) [28, 29] for summative assessment of competencies. Two of 10 stations in a circuit of tasks to be examined will concern randomly selected ultrasonography EPAs. Rating will follow the international multispecialty consensus on the content of a generic ultrasound rating scale [29]. However, in addition, students will receive qualitative real-time feedback on their performance at every single station. As recommended for assessment of workplace-based competencies, in both DOPS and OSCE, observation and assessment will happen simultaneously. Slower learners may need multiple attempts to meet requirements. Students who successfully passed OSCE will receive an official certificate from the German Ultrasound Society.

During the final year, portfolio assessment will be applied. Students shall keep a logbook and complete entries concerning listed skills and competencies. Expected entrustment level regarding EPAs is direct pro-active supervision and, however, should be enhanced to indirect reactive supervision for entry into residency. Supervisors should confirm successfully demonstrated EPAs in the logbook. In addition, they are encouraged to provide feedback on student’s performance over the entire period of the final year, preferably through an ongoing dialog (Table 1).

For evaluation of the curriculum itself, student and supervisor surveys are scheduled following every training level to obtain feedback results. Quality control will also include achieved OSCE scores and resource requirements. The curriculum shall always be open to improvement and development.

## Limitation

As there is considerable heterogeneity across universities and teaching hospitals, participating clinical departments and POCUS topics may vary according to particularities of each institution. Finally, to date, first students who pass through the curriculum are only on the second of four training levels. Therefore, evaluation is not yet completed.

## Conclusion

Ultrasonography gains increasing acceptance in most medical specialties. This is largely due to its high degree of utility and safety. Benefit of structured ultrasonography training is broadly recognized, but curricula are rarely implemented [30]. We present a concept for a longitudinal, competency-based compulsory curriculum that integrates ultrasonography into preclinical and clinical education to train a new generation of physicians. In addition, the curriculum aims to enhance self-determined learning and mutual benefits regarding learning success in disciplines involved. Required small-group “probe-in-the-hand” training probably will increase personnel and financial burden. However, costs can be saved by e-learning and inclusion of student tutors. As participating departments share benefits arising from integration of ultrasonography, they may contribute to the costs proportionally. Overall, with early access to continuous “probe-in-the-hand” training in medical education, ultrasonography could be seen as an extension of physician’s senses like the stethoscope has long since been [23]. In daily patient care, high-level ultrasonography will be regarded as a matter of course.

## Supplementary Information

**Additional file 1: Table S1** Literature Review on Established Ultrasonography Curricula. **Table S2** Entrustable Professional Activity: Focused Assessment with Sonography for Trauma (FAST). **Table S3** Entrustable Professional Activity: Focused Cardiac Ultrasonography (FOCUS). **Table S4** Entrustable Professional Activity: Bedside Lung Ultrasonography in Emergencies (BLUE)/Fluid Administration Limited by Lung Sonography (FALLS). **Table S5** Entrustable Professional Activity: Focused Sonography Abdomen. **Table S6** Entrustable Professional Activity: Focused Ultrasonography of Lower Extremity Deep Veins. **Table S7** Entrustable Professional Activity: Ultrasonography-Guided Peripheral Intravenous Insertion.

## Data Availability

Data sharing is not applicable to this article as no datasets were generated or analyzed during the current study.

## Abbreviations

DOPS: Direct observation of procedural skills
EPA: Entrustable professional activity
ILO: Intended learning outcome
OSCE: Objective structured clinical examination
POCUS: Point-of-care ultrasonography
US: Ultrasonography

## References

[1] Moore CL, Copel JA (2011). Point-of-care ultrasonography. N Engl J Med.

[2] Dinh VA, Lakoff D, Hess J (2016). Medical student core clinical ultrasound milestones: a consensus among directors in the United States. J Ultrasound Med.

[3] Hoffmann B, Blaivas M, Abramowicz J (2020). Medical student ultrasound education, a WFUMB position paper, Part II. A consensus statement of ultrasound societies EFSUMB statement on medical student education in ultrasound [long version]. Med Ultrason.

[4] Cantisani V, Dietrich CF, Badea R (2016). EFSUMB statement on medical student education in ultrasound [long version]. Ultrasound Int Open.

[5] Vallée A, Blacher J, Cariou A, Sorbets E (2020). Blended learning compared to traditional learning in medical education: systematic review and meta-analysis. J Med Internet Res.

[6] Vavasseur A, Muscari F, Meyrignac O (2020). Blended learning of radiology improves medical students' performance, satisfaction, and engagement. Insights Imaging.

[7] Thomas AT, Kern DE, Hughes MT, Chen BY (1998). Curriculum development for medical education: a six step approach.

[8] Bahner DP, Goldman E, Way D, Royall NA, Liu YT (2014). The state of ultrasound education in U.S. Medical Schools: results of a national survey. Acad Med.

[9] Prosch H, Radzina M, Dietrich CF (2020). Ultrasound curricula of student education in Europe: summary of the experience. Ultrasound Int Open.

[10] Biggs J, Tang C (2011). Teaching for quality learning at university.

[11] Celebi N, Zwirner K, Lischner U (2012). Student tutors are able to teach basic sonographic anatomy effectively: a prospective randomized controlled trial. Ultraschall Med.

[12] Ten Cate O, Durning S (2007). Dimensions and psychology of peer teaching in medical education. Med Teach.

[13] Harden RM (1999). What is a spiral curriculum?. Med Teach.

[14] Ten Cate O, Taylor DR (2020). The recommended description of an entrustable professional activity: AMEE Guide No. 140. Med Teach.

[15] Kennedy TJ, Regehr G, Baker GR, Lingard L (2008). Point-of-care assessment of medical trainee competence for independent clinical work. Acad Med.

[16] Teichgräber UK, Meyer JM, Poulsen Nautrup C, von Rautenfeld DB (1996). Ultrasound anatomy: a practical teaching system in human gross anatomy. Med Educ.

[17] Brown B, Adhikari S, Marx J, Lander L, Todd GL (2012). Introduction of ultrasound into gross anatomy curriculum: perceptions of medical students. J Emerg Med.

[18] Fraser S, Wright AD, van Donkelaar P, Smirl JD (2019). Cross-sectional comparison of spiral versus block integrated curriculums in preparing medical students to diagnose and manage concussions. BMC Med Educ.

[19] Frank JR, Mungroo R, Ahmad Y, Wang M, De Rossi S, Horsley T (2010). Toward a definition of competency-based education in medicine: a systematic review of published definitions. Med Teach.

[20] Kobal SL, Trento L, Baharami S (2005). Comparison of effectiveness of hand-carried ultrasound to bedside cardiovascular physical examination. Am J Cardiol.

[21] Hoppmann RA, Rao VV, Bell F (2015). The evolution of an integrated ultrasound curriculum (iUSC) for medical students: 9-year experience. Crit Ultrasound J.

[22] Teichgräber U, Ingwersen M, Bürckenmeyer F (2021). Structured work-based learning in undergraduate clinical radiology immersion experience. BMC Med Educ.

[23] Solomon SD, Saldana F (2014). Point-of-care ultrasound in medical education–stop listening and look. N Engl J Med.

[24] Holzhausen Y, Maaz A, Renz A, Bosch J, Peters H (2019). Development of entrustable professional activities for entry into residency at the Charité Berlin. GMS J Med Educ.

[25] Rempell JS, Saldana F, DiSalvo D (2016). Pilot point-of-care ultrasound curriculum at Harvard Medical School: early experience. West J Emerg Med.

[26] Mullen A, Kim B, Puglisi J, Mason NL (2018). An economical strategy for early medical education in ultrasound. BMC Med Educ.

[27] Biggs J (1996). Enhancing teaching through constructive alignment. High Educ.

[28] Harden RM, Gleeson FA (1979). Assessment of clinical competence using an objective structured clinical examination (OSCE). Med Educ.

[29] Tolsgaard MG, Todsen T, Sorensen JL (2013). International multispecialty consensus on how to evaluate ultrasound competence: a Delphi consensus survey. PLoS One.

[30] Wolf R, Geuthel N, Gnatzy F, Rotzoll D (2019). Undergraduate ultrasound education at German-speaking medical faculties: a survey. GMS J Med Educ.

