# Supplementary material for: Integration of ultrasonography training into undergraduate medical education: catch up with professional needs
Source: Insights Imaging. 2022 Sep 24;13:150. doi: 10.1186/s13244-022-01296-3 (PMC9509508; doi:10.1186/s13244-022-01296-3)
Supplement: Supplementary file 1 — Additional file 1: Table S1 Literature Review on Established Ultrasonography Curricula. Table S2 Entrustable Professional Activity: Focused Assessment with Sonography for Trauma (FAST). Table S3 Entrustable Professional Activity: Focused Cardiac Ultrasonography (FOCUS). Table S4 Entrustable Professional Activity: Bedside Lung Ultrasonography in Emergencies (BLUE)/Fluid Administration Limited by Lung Sonography (FALLS). Table S5 Entrustable Professional Activity: Focused Sonography Abdomen. Table S6 Entrustable Professional Activity: Focused Ultrasonography of Lower Extremity Deep Veins. Table S7 Entrustable Professional Activity: Ultrasonography-Guided Peripheral Intravenous Insertion. [file 13244_2022_1296_MOESM1_ESM.pdf]

ELECTRONIC SUPPLEMENTARY MATERIAL

Integration of Ultrasonography Training into Undergraduate Medical Education: Catch up With Professional Needs

Content

Table 1	Literature Review on Established Ultrasonography Curricula
Table 2	Entrustable Professional Activity: Focused Assessment with Sonography for Trauma (FAST)
Table 3	Entrustable Professional Activity: Focused Cardiac Ultrasonography (FOCUS)
Table 4	Entrustable Professional Activity: Bedside Lung Ultrasonography in Emergencies (BLUE)/Fluid Administration Limited by Lung Sonography (FALLS)
Table 5	Entrustable Professional Activity: Focused Sonography Abdomen
Table 6	Entrustable Professional Activity: Focused Ultrasonography of Lower Extremity Deep Veins
Table 7	Entrustable Professional Activity: Ultrasonography-Guided Peripheral Intravenous Insertion
References	

Supplemental Table 1 Literature review on established ultrasonography curricula

Reference	Date estab-lished	Stated goals	Mandatory vs. elective	Phase of training	Program length	Program type	Assessment	Outcomes reported
L Trembley et al. (Pittsburg, PA, USA) 2020[1]	2018	Distinguish between normal and pathologic sonographic findings<br><br>Identify pathologies	Elective	Preclinical	4 weeks	US in correlation with physical exam”<br><br>90-min weekly sessions: brief introduction and scanning practice (3-4 students per group)<br><br>Adjunct of clinical pathology: 30 min lectures created by an emergency medicine resident	None	Online survey
N Celebi (Waiblingen, Germany) 2019[2]	2016/ 2017	To ensure quality in the provision of ultrasound examinations<br><br>Curriculum for theoretical knowledge and a limited number of essential skills	Incorporated in the mandatory internship of internal medicine	Clinical (5th year)	90 min lecture<br><br>12.5 h hands-on course	• Lecture/Script<br>• Hands-on: Peer-teaching (1 tutor/4 students, 3 sessions)	• Multiple choice questions<br>• OSCE<br>(Final examination)<br><br>Student satisfaction: mandatory online evaluation every term	Student satisfaction (pre-test: elective course for 27 students)

(continued)

Literature review on established ultrasonography curricula (continued)

Reference	Date estab-lished	• Stated goals	Mandatory vs. elective	Phase of training	Program length	Program type	Assessment	Outcomes reported
JS Rempell et al. (Boston, MA, USA) 2016[3]	2013/ 2014	To gain attitude, understanding, and knowledge of US	Mandatory and elective advanced session (3h)	Preclinical (1st and 2nd year)	2 years	Integration of US into existing curriculum:<br>• 1st year: 4 hands-on sessions during gross anatomy over a 3-month period)<br>• 2nd year: during physical exam course (four 4h-sessions)	Survey on knowledge: basic questions on physics and views	Early experience: Pre- and post-curriculum survey
J Minardi et al. (Morgantown, VA, USA) 2019[4]	2013	To teach high-yield ultrasound content with broad relevance across most medical specialties	Mandatory and elective components	Preclinical and clinical	4 years	Longitudinal US curricular “thread”<br><br>Curricular components in many courses and clerkship	Written and practical competency assessment from the onset	Survey 2016 (optional, anonymous)
G Serrao et al. (Milan, Italy) 2017[5]	2009	Training future physicians to prepare them for their professional life	Not specified	Preclinical (1st year)	1 year	Vertical integration of US with anatomy<br><br>Peer tutors	Assignment of skillfulness scores to students	Scores:<br>• peer tutors vs. learners<br>• Women vs. men<br>• 3 modules (musculoskeletal, heart, abdomen/pelvis)

(continued)

Literature review on established ultrasonography curricula (continued)

Reference	Date established	• Stated goals	Mandatory vs. elective	Phase of training	Program length	Program type	Assessment	Outcomes reported
A Hoppmann (Columbia, SC, USA) 2011[6], 2015[7]	2006	To enhance medical education<br><br>To train students conducting focused US examinations<br><br>To improve patient safety and quality of care	Required and voluntary laboratory sessions	Preclinical and clinical	4 years	Horizontally and vertically integrated (in conjunction with anatomy, physiology, clinical medicine)<br><br>Competency based<br><br>• Lectures/ demonstrations<br>• Hands-on sessions<br>• Web-based learning modules	Formative and summative<br><br>• Image interpretation /feedback<br>• OSCEs<br>• Multiple choice examination<br><br>Assessment concerns both US and patient interaction	9 years<br><br>• Anonymous course evaluation by students<br>• Recommendations for starting US programs
S Rao et al. (Detroit, MI, USA) 2008[8]	2006/ 2007	To familiarize students with the US machine<br><br>To provide a foundation for US use in clinical years	Elective	Preclinical (1st year)	1 year	Six 90-min organ-system sessions<br><br>Lecture, hands-on, and clinical correlation components	Faculty assessment of student skills:<br>9-item dichotomous check sheet	Evaluation:<br>• Overall experience with the curriculum<br>• Technical skills

(continued)

Literature review on established ultrasonography curricula (continued)

Reference	Date established	Stated goals	Mandatory vs. elective	Phase of training	Program length	Program type	Assessment	Outcomes reported
DP Bahner et al., (Columbus, OH, USA) 2013[9, 10]	2005/ 2006	To reduce educational burden for physician residency programs<br><br>To improve overall physician competency	Mandatory/ Elective	Clinical (4th year)<br><br>Based on 3 years training incl. pre-clinical	1 year<br><br>(3 pre-ceding years)	Advanced training program (based on a vertical program starting in the first year)<br><br>• One-hour online module<br>• Didactic instruction (2h lecture)<br>• Journal club (student-led)<br>• Hands-on training (twice per months)<br>• Acting as teacher and patient (10h)<br>• Final project	• Monthly quizzes<br>• Final practical and written exams	Student survey
A Mullen et al. (Elk Grove, CA, USA) 2018[11]	Not speci-fied	To provide a temporally and fiscally economical US training	Elective	Preclinical (1st year)	4 weeks<br><br>Six 30-min hands-on sessions	• Small groups (4-6 students)<br>• Courses held by a single faculty member with<br>• one US machine<br>• (28 students)	• Pre-/post-training skill evaluation surveys<br>• Numerical scores by instructor during real-time evaluation of performance	Participant survey<br>Instructor scoring

(continued)

Literature review on established ultrasonography curricula (continued)

Reference	Date estab-lished	Stated goals	Mandatory vs. elective	Phase of training	Program length	Program type	Assessment	Outcomes reported
JY Fu et al. (Loma Linda, CA, USA) 2016[12]	Not speci-fied	To equip learners with confidence to apply US	Mandatory and elective	Preclinical and clinical	4 years	Vertical integration of US into the medical curriculum<br>• Brief handout<br>• Short prequiz<br>• Laboratory sessions<br><br>Peer-taught sessions	1st year: US OSCE	OSCE

US = ultrasonography, OSCE = objective structured clinical examination, POCUS = point-of-care ultrasonography.

Supplemental Table 2 Entrustable professional activity: focused assessment with sonography for trauma (FAST)

**Title**	**Ultrasonography scanning of traumatized patients**
Specification and limitations	Performing and interpreting ultrasonography in traumatized patients in the emergency department to detect or rule out abdominal and pericardial free fluid.<br><br>Limitations<br>• POCUS course: learners scan each other (image acquisition and identification)<br>• Skills lab: using simulation of pathological findings<br>• Internship: scanning on patients
Knowledge, skills, attitude, and experience	Knowledge and skills:<br>Recognition of appropriate indication of point-of-care ultrasonography (POCUS)<br><br><u>Trauma pathology recognition</u><br>Pericardial effusion<br>Peritoneal free fluid assessment<br>Attitude:<br>Appropriate patient interaction<br>Appropriate infection control practices<br>Compliance to confidentiality standards<br>Ability to communicate and work effectively with others<br>Recognize scope, limitations, and when to ask for help<br>Experience:<br>Online learning video (5 minutes)<br>POCUS course (1 teaching unit*, small groups†)<br>Skills lab (1/2 teaching unit, small groups, peer teachers)<br>Scanning on patients during sub-internship
Most relevant competency domains	Medical expert, communicator, collaborator, professional
Information sources to assess progress and support summative entrustment	• Objective structured clinical examination (OSCE) for entry into sub-internship<br>• Direct observation of procedural skills (DOPS) during sub-internship
Expected entrustment/supervision level at which stage of training‡	• Direct, pro-active supervision (level 2) for entry into sub-internship.<br>• Indirect, reactive supervision (level 3) for entry into residency

*A teaching unit equals 45 minutes
†Maximum group size of 6 learners per supervisor
‡According to Ten Cate [13]: level 2: the learner is allowed to execute the EPA with direct, pro-active supervision, present in the room; level 3: the learner is allowed to carry out the EPA without a supervisor in the room, but quickly available if needed

Supplemental Table 3 Entrustable professional activity: focused cardiac ultrasonography (FOCUS)

Title	Echocardiography
Specification and limitations	Performing and interpreting cardiac ultrasonography in patients with chest pain, dyspnea, and/or shock of unknown origin.<br><br>Limitations<br>• POCUS course: learners scan each other (image acquisition and identification)<br>• Skills lab: using simulation of pathological findings<br>• Internship: scanning on patients
Knowledge, skills, attitude, and experience	Knowledge and skills:<br>Recognition of appropriate indication of point-of-care ultrasonography (POCUS)<br><br>Image acquisition and identification<br>Cardiac valves, cardiac apex, pericardium<br>Subcostal 4-chamber view<br>Apical 4-chamber view<br>Parasternal long-axis view<br>Parasternal short-axis view<br><br>Cardiac pathology recognition:<br>Gross left ventricular function<br>Right ventricular overload/failure<br>Pericardial effusion<br>Attitude:<br>Appropriate patient interaction<br>Appropriate infection control practices<br>Compliance to confidentiality standards<br>Ability to communicate and work effectively with others<br>Recognize scope, limitations, and when to ask for help<br>Experience:<br>Online learning video (5 minutes)<br>POCUS course (1 teaching unit*, small groups†)<br>Skills lab (1/2 teaching unit, small groups, peer teachers)<br>Scanning on patients during sub-internship
Most relevant competency domains	Medical expert, communicator, collaborator, professional
Information sources to assess progress and support summative entrustment	• Objective structured clinical examination (OSCE) for entry into sub-internship<br>• Direct observation of procedural skills (DOPS) during sub-internship
Expected entrustment/supervision level at which stage of training‡	• Direct, pro-active supervision (level 2) for entry into sub-internship.<br>• Indirect, reactive supervision (level 3) for entry into residency

*A teaching unit equals 45 minutes
†Maximum group size of 6 learners per supervisor
‡According to Ten Cate [13]: level 2: the learner is allowed to execute the EPA with direct, pro-active supervision, present in the room; level 3: the learner is allowed to carry out the EPA without a supervisor in the room, but quickly available if needed

Supplemental Table 4 Entrustable professional activity: bedside lung ultrasonography in emergencies (BLUE)/fluid administration limited by lung sonography (FALLS)

**Title**	**Thoracic ultrasonography scanning**
Specification and limitations	Performing and interpreting thoracal ultrasonography in patients with respiratory insufficiency and/or dyspnea of unknown origin.<br><br>Limitations<br>• POCUS course: learners scan each other (image acquisition and identification)<br>• Skills lab: using simulation of pathological findings<br>• Internship: scanning on patients
Knowledge, skills, attitude, and experience	Knowledge and skills:<br>Recognition of appropriate indication of point-of-care ultrasonography (POCUS)<br><br>Image acquisition and identification<br>Pleura<br>Rib shadows<br>Lung sliding (B-mode, M-mode)<br>Diaphragm<br>A lines<br>B lines<br><br>Thoracic pathology recognition:<br>Pneumothorax (in addition to FAST: eFAST)<br>Pleural effusion<br>Consolidation<br>Attitude:<br>Appropriate patient interaction<br>Appropriate infection control practices<br>Compliance to confidentiality standards<br>Ability to communicate and work effectively with others<br>Recognize scope, limitations, and when to ask for help<br>Experience:<br>Online learning video (5 minutes)<br>POCUS course (1 teaching unit*, small groups†)<br>Skills lab (1/2 teaching unit, small groups, peer teachers)<br>Scanning on patients during sub-internship
Most relevant competency domains	Medical expert, communicator, collaborator, professional
Information sources to assess progress and support summative entrustment	• Objective structured clinical examination (OSCE) for entry into sub-internship<br>• Direct observation of procedural skills (DOPS) during sub-internship
Expected entrustment/supervision level at which stage of training‡	• Direct, pro-active supervision (level 2) for entry into sub-internship.<br>• Indirect, reactive supervision (level 3) for entry into residency

*A teaching unit equals 45 minutes
†Maximum group size of 6 learners per supervisor

‡According to Ten Cate [13]: level 2: the learner is allowed to execute the EPA with direct, pro-active supervision, present in the room; level 3: the learner is allowed to carry out the EPA without a supervisor in the room, but quickly available if needed

Supplemental Table 5 Entrustable professional activity: focused sonography abdomen

**Title**	**Abdominal ultrasonography scanning**
Specification and limitations	Performing and interpreting abdominal ultrasonography to detect or rule out pathological organ alterations and free fluid.<br><br>Limitations<br>• POCUS course: learners scan each other (image acquisition and identification)<br>• Skills lab: using simulation of pathological findings<br>• Internship: scanning on patients
Knowledge, skills, attitude, and experience	Knowledge and skills:<br>Recognition of appropriate indication of point-of-care ultrasonography (POCUS)<br><br><u>Image acquisition and identification</u><br>Liver<br>Spleen<br>Aorta<br>Gallbladder<br>Urinary bladder<br>Kidneys<br>Uterus<br>Small and large intestines<br><br><u>Abdominal pathology recognition:</u><br>Cholelithiasis<br>Peritoneal free fluid assessment<br>Hydronephrosis<br>Qualitative bladder volume assessment<br>Abdominal aortic aneurysm<br>Attitude:<br>Appropriate patient interaction<br>Appropriate infection control practices<br>Compliance to confidentiality standards<br>Ability to communicate and work effectively with others<br>Recognize scope, limitations, and when to ask for help<br>Experience:<br>Online learning video (5 minutes)<br>POCUS course (1 teaching unit*, small groups†)<br>Skills lab (1/2 teaching unit, small groups, peer teachers)<br>Scanning on patients during sub-internship
Most relevant competency domains	Medical expert, communicator, collaborator, professional
Information sources to assess progress and support summative entrustment	• Objective structured clinical examination (OSCE) for entry into sub-internship<br>• Direct observation of procedural skills (DOPS) during sub-internship

Expected entrustment/supervision level at which stage of training‡	• Direct, pro-active supervision (level 2) for entry into sub-internship.<br>• Indirect, reactive supervision (level 3) for entry into residency

*A teaching unit equals 45 minutes
†Maximum group size of 6 learners per supervisor
‡According to Ten Cate [13]: level 2: the learner is allowed to execute the EPA with direct, pro-active supervision, present in the room; level 3: the learner is allowed to carry out the EPA without a supervisor in the room, but quickly available if needed

Supplemental Table 6 Entrustable professional activity: focused ultrasonography of lower extremity deep veins

**Title**	**Ultrasonography scanning of lower extremity deep veins**
Specification	Performing and interpreting lower extremity vein ultrasonography.<br><br>Limitations<br>• POCUS course: learners scan each other (image acquisition and identification)<br>• Skills lab: using simulation of pathological findings<br>• Internship: scanning on patients
Knowledge, skills, attitude, and experience	Knowledge and skills:<br>Recognition of appropriate indication of point-of-care ultrasonography (POCUS)<br><br><u>Image acquisition and identification</u><br>Common femoral and popliteal veins<br><br><u>Vascular pathology recognition:</u><br>Deep vein thrombosis assessment<br>Attitude:<br>Appropriate patient interaction<br>Appropriate infection control practices<br>Compliance to confidentiality standards<br>Ability to communicate and work effectively with others<br>Recognize scope, limitations, and when to ask for help<br>Experience:<br>Online learning video (5 minutes)<br>POCUS course (1 teaching unit*, small groups†)<br>Skills lab (1/2 teaching unit, small groups, peer teachers)<br>Scanning on patients during sub-internship
Most relevant competency domains	Medical expert, communicator, collaborator, professional
Information sources to assess progress and support summative entrustment	• Objective structured clinical examination (OSCE) for entry into sub-internship<br>• Direct observation of procedural skills (DOPS) during sub-internship

Expected entrustment/supervision level at which stage of training‡	• Direct, pro-active supervision (level 2) for entry into sub-internship.<br>• Indirect, reactive supervision (level 3) for entry into residency

*A teaching unit equals 45 minutes
†Maximum group size of 6 learners per supervisor
‡According to Ten Cate [13]: level 2: the learner is allowed to execute the EPA with direct, pro-active supervision, present in the room; level 3: the learner is allowed to carry out the EPA without a supervisor in the room, but quickly available if needed

Supplemental Table 7 Entrustable professional activity: ultrasonography-guided peripheral intravenous insertion

**Title**	**Inserting a central venous catheter using ultrasonography guidance**
Specification and limitations	Achieving ultrasonography-guided central venous access using a peripherally inserted central catheter (PICC) and an internal jugular central venous catheter (CVC).<br><br>Limitations<br>• POCUS course: ultrasonography imaging of lower and upper arm veins and jugular veins in peers<br>• Skills lab: ultrasonography-guided central venous cannulation on training phantoms<br>• Internship: performing ultrasonography-guided central venous access in patients
Knowledge, skills, attitude, and experience	Knowledge and skills:<br>Image acquisition and identification<br>Internal jugular, subclavian, cephalic, basilic, and brachial veins<br><br>Ultrasonography-guided central venous access<br>Attitude:<br>Appropriate patient interaction<br>Appropriate infection control practices<br>Compliance to confidentiality standards<br>Ability to communicate and work effectively with others<br>Recognize scope, limitations, and when to ask for help<br>Experience:<br>Online learning video (5 minutes)<br>POCUS course (1 teaching unit*, small groups†)<br>Skills lab (1/2 teaching unit, small groups, peer teachers)<br>Scanning on patients during sub-internship
Most relevant competency domains	Medical expert, communicator, collaborator, professional
Information sources to assess progress and support summative entrustment	• Objective structured clinical examination (OSCE) for entry into sub-internship<br>• Direct observation of procedural skills (DOPS) during sub-internship

Expected entrustment/supervision level at which stage of training‡	• Direct, pro-active supervision (level 2) for entry into sub-internship.<br>• Indirect, reactive supervision (level 3) for entry into residency

*A teaching unit equals 45 minutes
†Maximum group size of 6 learners per supervisor
‡According to Ten Cate [13]: level 2: the learner is allowed to execute the EPA with direct, pro-active supervision, present in the room; level 3: the learner is allowed to carry out the EPA without a supervisor in the room, but quickly available if needed

References

1 Trembley L, Radomski M (2020) Use of Ultrasound in Introducing Anatomical Pathology to Preclinical Medical Students, in Correlation with Physical Exam Curricula. MedEdPORTAL 16:10950

2 Celebi N, Griewatz J, Malek NP et al (2019) Development and implementation of a comprehensive ultrasound curriculum for undergraduate medical students - a feasibility study. BMC Med Educ 19:170

3 Rempell JS, Saldana F, DiSalvo D et al (2016) Pilot Point-of-Care Ultrasound Curriculum at Harvard Medical School: Early Experience. West J Emerg Med 17:734-740

4 Minardi J, Ressetar H, Foreman T et al (2019) Longitudinal Ultrasound Curriculum Incorporation at West Virginia University School of Medicine: A Description and Graduating Students' Perceptions. J Ultrasound Med 38:63-72

5 Serrao G, Tassoni M, Magenta-Biasina AM et al (2017) Virtual Dissection by Ultrasound: Probe Handling in the First Year of Medical Education. Ultrasound Int Open 3:E156-E162

6 Hoppmann RA, Rao VV, Poston MB et al (2011) An integrated ultrasound curriculum (iUSC) for medical students: 4-year experience. Crit Ultrasound J 3:1-12

7 Hoppmann RA, Rao VV, Bell F et al (2015) The evolution of an integrated ultrasound curriculum (iUSC) for medical students: 9-year experience. Crit Ultrasound J 7:18

8 Rao S, van Holsbeeck L, Musial JL et al (2008) A pilot study of comprehensive ultrasound education at the Wayne State University School of Medicine: a pioneer year review. J Ultrasound Med 27:745-749

9 Bahner DP, Royall NA (2013) Advanced ultrasound training for fourth-year medical students: a novel training program at The Ohio State University College of Medicine. Acad Med 88:206-213

10 Prats MI, Royall NA, Panchal AR et al (2016) Outcomes of an Advanced Ultrasound Elective: Preparing Medical Students for Residency and Practice Advanced ultrasound training for fourth-year medical students: a novel training program at The Ohio State University College of Medicine. J Ultrasound Med 35:975-982

11 Mullen A, Kim B, Puglisi J, Mason NL (2018) An economical strategy for early medical education in ultrasound. BMC Med Educ 18:169

12 Fu JY, Krause C, Krause R et al (2016) Integration of Point-of-Care Ultrasound Training into Undergraduate Medical Curricula--A Perspective from Medical Students. J Med Educ Curric Dev 3

13 Ten Cate O, Taylor DR (2020) The recommended description of an entrustable professional activity: AMEE Guide No. 140. Med Teach. 10.1080/0142159x.2020.1838465:1-9
